# Benign cystic mesothelioma of the appendix presenting in a woman: a case report

**DOI:** 10.1186/1752-1947-4-394

**Published:** 2010-12-03

**Authors:** Donal B O'Connor, David Beddy, Muyiwa A Aremu

**Affiliations:** 1 Department of Surgery, St Vincent's University Hospital, Elm Park, Dublin 4, Ireland

## Abstract

**Introduction:**

Benign cystic mesothelioma or peritoneal inclusion cysts are rare benign abdominal tumors usually occurring in females of reproductive age. These cysts present as abdominopelvic pain or masses but are often found on imaging or incidentally at surgery. They are commonly associated with pelvic inflammatory disease, endometriosis, or ovarian cysts. We report what is, to the best of our knowledge, the first case of a benign cystic mesothelioma complicating a presentation of acute appendicitis.

**Case Presentation:**

A 19-year-old Irish Caucasian woman presented with abdominal pain. Imaging suggested appendicitis with abscess formation. She was treated with antibiotics and scheduled for interval appendicectomy. At laparoscopy, an unusual cystic mass was found arising from the appendix. Histology revealed benign cystic mesothelioma.

**Conclusion:**

We report what is, to the best of our knowledge, the first case of a benign cystic mesothelioma arising from the appendix and complicating a presentation of acute appendicitis. This is a benign pathology, but recurrences are not uncommon. Benign cystic mesothelioma should be included in the differential when investigating pelvic masses or abscesses associated with either appendicitis or pelvic inflammatory disease in women.

## Introduction

Benign cystic mesothelioma (BCM) or peritoneal inclusion cysts are rare abdominal tumors usually occurring in women of reproductive age. These cysts present as abdominal or pelvic pain or masses but are often found on imaging or incidentally at surgery. There have been many cases described associated with pelvic inflammatory disease, endometriosis, or ovarian cysts. We describe the first case of a benign cystic mesothelioma arising from the appendix and complicating a presentation of acute appendicitis.

## Case Presentation

A 19-year-old Irish Caucasian woman presented to the hospital with a three-day history of abdominal pain and fever. The pain was gradual in onset and associated with nausea and one episode of vomiting. She had no urinary symptoms, and her last menstrual period had finished the previous day. She had no surgical history, and her medical history was significant only for viral meningitis two years previously. She denied any history of sexually transmitted disease or recent urinary tract infection. She was not taking regular medications and had no allergies. On examination, her vital signs were normal except for mild pyrexia of 37.4 °C. Examination of the abdomen revealed a tender mass in the right iliac fossa.

Laboratory investigations included a white cell count of 10,500 cells/mm, hemoglobin of 13.3 g/dl, and platelets of 212,000/mm. Urea and electrolytes were within normal ranges. Urine analysis was negative for leucocytes and urinary βHCG was negative.

**Figure 1:**
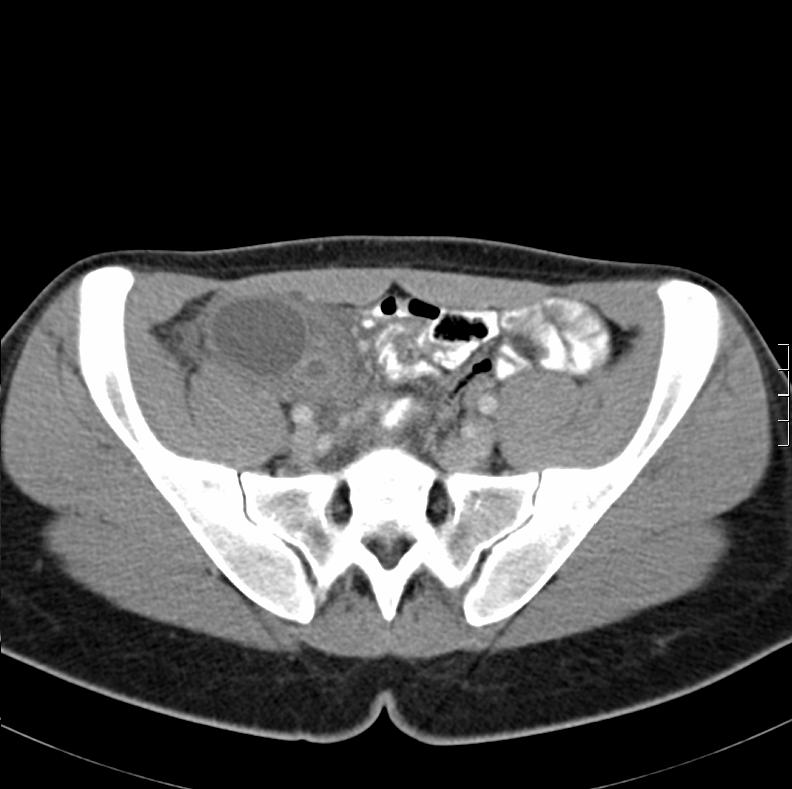
**Axial CT image showing a loculated cystic mass in the right pelvis which appears to contain the tip of the appendix**.

A computed tomography (CT) scan of the abdomen and pelvis was requested and showed a 10.4 × 4.5 × 3.8 cm loculated cystic mass in the right pelvis that appeared to contain the tip of the appendix (Figure 1). The patient remained febrile. Clinically, we made a working diagnosis of an appendix mass but considered a tubo-ovarian abscess as a differential. The patient was treated with intravenous antibiotics, and radiological drainage of the abscess was arranged. An ultrasound-guided drain was placed in the largest locule via the right iliac fossa. Unusually, 30 ml of serous fluid but no pus was aspirated. The drain was removed after three days with no further output. Drained fluid was sent for culture, and peripheral blood cultures showed no growth after 72 hours of incubation. After five days, intravenous antibiotics the patient was clinically well. She was discharged and readmitted two weeks later for an interval diagnostic laparoscopy, as we were now suspicious of a non-infective pathology based on the drain output. The patient consented to an appendicectomy if no other pathology was found.

**Figure 2:**
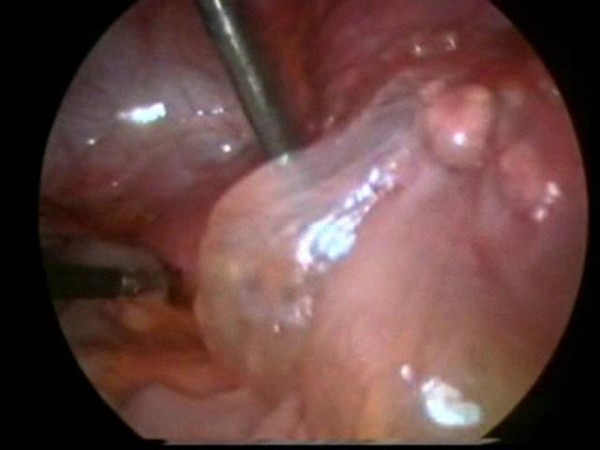
**Laparoscopy**. Operative photograph showing thin-walled cystic mass in the right iliac fossa above the appendix.

At laparoscopy, a multiloculated, thin-walled and translucent cystic mass was seen in the right iliac fossa (Figure 2). Adherent to the cystic mass was a spherical, smooth-walled cyst in continuity with the tip of the appendix. The rest of the appendix, caecum, and large and small bowel appeared grossly normal. Both ovaries and the uterus were visualized and found to be normal. The diagnosis was not clear at this point, but our differential included a mucinous cystadenoma or adenocarcinoma (pseudomyxoma peritonei). The lesion appeared very friable, and we were concerned we would rupture it and contaminate the pelvis with the cyst fluid. We made a decision to convert to an open procedure using a Lanz incision to safely perform an appendicectomy and remove the cystic mass. The incision incorporated the previous drain site.

**Figure 3:**
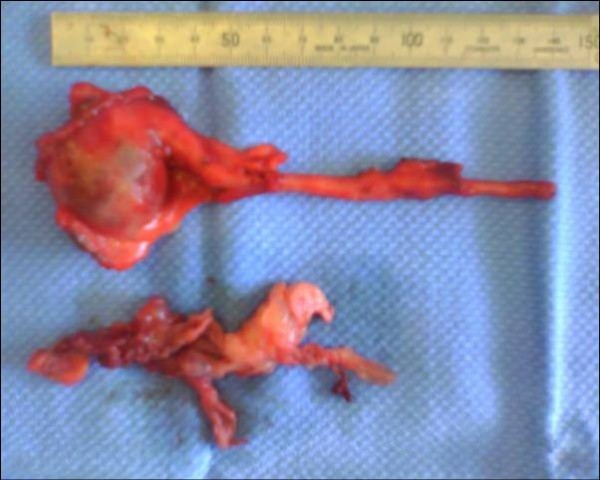
**Gross specimen of a 4 × 3 × 3 cm thick-walled cyst seen in continuity with the tip of the appendix**. Immediately below the 15-cm ruler in the photograph. Membranes of the remainder of the multiloculated cyst after removal from the appendix are seen lying toward the bottom of the photograph. The cyst had ruptured in transit from the operating table to the specimen photography table in the operating room.

**Figure 4:**
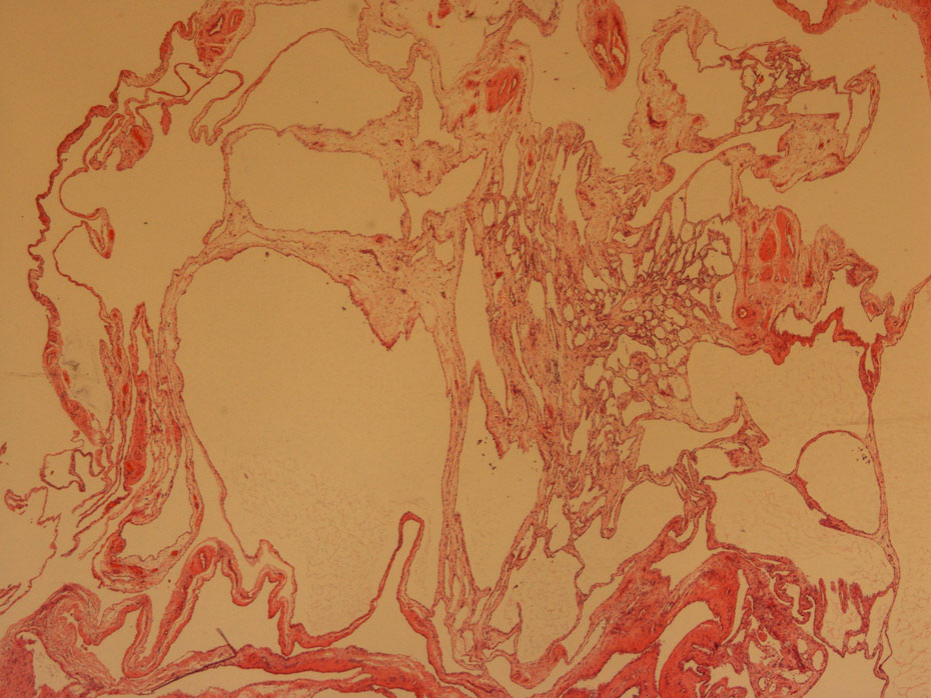
**Histology**. Microscopy showing cysts lined with flattened mesothelial cell and the walls composed of loose connective tissue with occasional chronic inflammatory cells.

Macroscopy showed a 12-cm appendix with an attached 4 × 4 × 3 cm smooth cyst containing clear fluid. Numerous smaller translucent cysts up to 0.7 cm in diameter were loosely attached to and easily separated from the larger cyst (Figure 3). We concluded that the radiological drain had entered one of these cysts. Histological analysis revealed cysts lined with flattened mesothelial cells, and the walls were composed of loose connective tissue with occasional chronic inflammatory cells (Figure 4). These findings were consistent with a histological diagnosis of a multiloculated benign cystic mesothelioma. The appendix showed resolving appendicitis with perforation at the tip. The patient was discharged well on the second postoperative day and was also well at six-week and three-month follow-up.

## Discussion

Benign cystic mesothelioma or peritoneal inclusion cysts are rare but well-described benign tumors of unknown etiology. First described by Plaut in 1928 (1), they are cystic mesothelial proliferations. They are thought to be due to an inflammatory reaction. They usually occur in the peritoneal cavity in the abdomen or pelvis, and the most common predisposing factors in the clinical history are previous surgery, pelvic inflammatory disease, or endometriosis. These conditions are believed to interfere with peritoneal reabsorption. This would tend to support a hypothesis of BCM being reactive and inflammatory rather than neoplastic (2). These conditions tend to occur in women of reproductive age, but cases have been reported in men (3). The most common sites are the serosal surfaces of the ovary and uterus, but cases outside the abdomen have been described, including the pleural cavity (4). Typical microscopic findings are a single layer of flattened mesothelial cells sometimes described as a hobnail configuration. Squamous metaplasia and papillae may also be seen (3).

The clinical presentation is usually abdominal or pelvic pain, a mass found clinically or radiologically, or an incidental surgical finding (5). BCM is considered to be a benign inflammatory process; however, malignant transformation has been reported (6). Diagnostic modalities include ultrasound and CT, but preoperative diagnosis is often not conclusive and there are no protocols for diagnostic imaging. The main differentials are ovarian cysts, ovarian tumors (benign or malignant), or cystic lymphangioma. When presenting acutely with signs of infection as in the case described here, pelvic inflammatory disease complicated by abscess would be the most common differential as associations with appendicitis are very rare.

Management currently involves surgical resection, but recurrences are well documented. There are no protocols for surgical management, and the literature is based on case reports and small case series. Laparoscopic resections have been described. While laparoscopy is an elegant tool for investigation of masses or pain in women, we believed open surgery to be safer when a malignant process was suspected owing to the possibility of cyst rupture and seeding. Follow-up after surgical resection includes clinical review and ultrasound or CT, but again there are no guidelines.

BCM involving the appendix is very rare. Only four other cases have been reported. Two were in middle-aged women presenting with abdominal pain and suspected appendicitis where cysts were found adjacent to but not involving the appendix (7, 8). In a third case, a BCM was found incidentally beside an otherwise unremarkable appendix at laparotomy for sigmoid diverticular disease (9). The other case involved a 28-year-old man who presented with appendicitis in which a 25-cm separate cystic mass was found (10). Our patient's case is unique in that the BCM was in direct continuity with the tip of the appendix and presented with both clinical signs and histological evidence of acute appendicitis.

## Conclusions

BCM is a rare benign tumor, but surgeons should include it in the differential when investigating abdominal masses or pain in women of reproductive age. This is the first reported case of a BCM arising from the appendix which complicated a presentation of acute appendicitis. Surgery is the authors' recommended treatment, but patients should be advised of the possibility of recurrence.

## Competing interests

The authors declare that they have no competing interests.

## Authors' contributions

DB O'C drafted and conceived the manuscript, DB assisted in the drafting and editing of the final manuscript, and MA performed critical revisions of the manuscript.

DB O'C and MA performed the operation. All authors read and approved the final manuscript.

## Consent

The authors have written informed consent from the patient for the publication in a medical journal of the manuscript and images. A copy of this consent can be made available to the editorial team.
